# An Integrated Mission Planning Framework for Sensor Allocation and Path Planning of Heterogeneous Multi-UAV Systems

**DOI:** 10.3390/s21103557

**Published:** 2021-05-20

**Authors:** Hongxing Zheng, Jinpeng Yuan

**Affiliations:** 1 School of Astronautics, Harbin Institute of Technology, Harbin 150001, China; 2 Institute of Manned Space System Engineering, China Academy of Space Technology, Beijing 100094, China; hit_yuan@sina.com

**Keywords:** heterogeneous multi-UAVs system, airborne sensor allocation, path planning, mission planning, two-level adaptive variable neighborhood search

## Abstract

Mission planning is the guidance for a UAV team to perform missions, which plays the most critical role in military and civil applications. For complex tasks, it requires heterogeneous cooperative multi-UAVs to satisfy several mission requirements. Meanwhile, airborne sensor allocation and path planning are the critical components of heterogeneous multi-UAVs system mission planning problems, which affect the mission profit to a large extent. This paper establishes the mathematical model for the integrated sensor allocation and path planning problem to maximize the total task profit and minimize travel costs, simultaneously. We present an integrated mission planning framework based on a two-level adaptive variable neighborhood search algorithm to address the coupled problem. The first-level is devoted to planning a reasonable airborne sensor allocation plan, and the second-level aims to optimize the path of the heterogeneous multi-UAVs system. To improve the mission planning framework’s efficiency, an adaptive mechanism is presented to guide the search direction intelligently during the iterative process. Simulation results show that the effectiveness of the proposed framework. Compared to the conventional methods, the better performance of planning results is achieved.

## 1. Introduction

Low cost, safety, robust endurance, and autonomous operation are essential advantages of unmanned aerial vehicles (UAVs), leading to widespread application in many fields. With the rapid development of UAVs, sensors, and communications technology, multiple heterogeneous UAVs perform complex tasks collaboratively has become an essential application paradigm. Due to its broad application in various practical, real-world missions (e.g., intelligence gathering, reconnaissance, and combat intelligence), the importance of multi-UAVs system mission planning is increasingly highlighted. In recent years, the mission planning of multi-UAV systems based on specific issues has been extensively studied. The mission planning for heterogeneous multi-UAVs systems, as an essential component of the conventional mission planning problem, focus on solving the integrated task assignment and path planning problem for heterogeneous multi-UAVs systems. Specifically, it aims to find an optimal solution to assign a reasonable mission route to each heterogeneous UAV to satisfy complex mission requirements and generate a corresponding flyable collision-free path.

Task assignment is an essential subproblem in mission planning, which belongs to the combinatorial optimization problem in operations research. For the multi-UAVs task assignment problem, it generally refers to assigning tasks to be performed for a multi-UAVs system, while meeting the task requirements, optimizing the given objectives, such as mission completion deadline, total fuel consumption, etc. The heterogeneous multi-UAVs system improves task execution efficiency, but it also increases the task assignment problem’s complexity. Due to the task assignment’s natural complexity, it belongs to a type of NP-hard combinatorial optimization problem, which makes the deterministic method unable to solve the problem in polynomial time [1]. Therefore, many heuristics methods have been studied for solving the task assignment problem to generate a suboptimal solution within the accepted time, such as self-organizing map neural network [2], genetic algorithm [3,4], ant colony algorithm [5], and particle swarm optimization [6].

Path planning is another essential subproblem in mission planning. Generally, path planning is to generate collision-free paths connecting the start and end positions in space. As a fundamental problem, path planning has been widely studied in many fields, such as robots, robotic arms, games, and UAVs. Path planning methods are mainly classified in terms of heuristic-based [7], learning-based [8,9,10], sampling-based [11], geometry-based [12], etc. Note that solving mission planning problems usually requires evaluating many solutions to obtain an optimized mission plan. Therefore, the path planning method incorporates into the mission planning framework is required to generate the optimal collision-free path as fast as possible. Generally, heuristic-based incorporating geometry-based applications are suitable for generating collision-free and flyable paths in the mission planning framework, which depends on its fast computing speed.

Task assignment and path planning problems are usually integrated into a unified framework, depending on the coupling of the two problems. Edison et al. [13] addressed the mission planning problem that multi-UAVs cooperatively execute a group of successive tasks with strict priority on each ground target. The mission planning issue is formulated as an integrated task assignment and path planning problem. Meanwhile, a mission planning framework based on a genetic algorithm is developed to solve the problem. Deng et al. [14] presented an improved genetic algorithm with multi-type genes to solve the integrated task assignment and path planning problem. Further, a mirror representation of vehicles is developed to deal with the limited resource constraint. Wang et al. [15] presented a reconnaissance task assignment model to optimize UAVs’ task sequence by the opposition-based genetic algorithm using double-chromosomes encoding and multiple mutations. Meanwhile, heterogeneous targets classified according to features of target geometry and sensor’s field of view are addressed. Chen et al. [16] presented a systematical framework based on the modified two-part wolf pack search algorithm. In addition, the parameter and time-sensitive uncertainties are considered in the extended task assignment problem, which is formulated by a robust optimization method. Jia et al. [17] studied the mission planning problem with stochastic velocities and time windows for a heterogeneous multi-UAVs system, which is solved by a modified genetic algorithm. Further, a path extension mechanism based on the dubins method is proposed to meet the tasks’ priority constraints. Mufalli et al. [18] proposed a column generation algorithm to deal with integrated sensor selection and route planning for UAVs. Deng et al. [19] proposed a novel large-area-coverage planning framework that collectively optimizes the paths for aerial and ground vehicles under the background of multiple UAVs and a ground vehicle cooperative large area coverage mission. Causa et al. [20] presented an algorithm for solving multi-UAV path planning in scenarios with heterogeneous Global Navigation Satellite Systems (GNSS) coverage. Further, Causa et al. [21] tackled strategic path planning for a multi-UAV routing problem in low altitude urban environment. A multi-step strategy was presented, which included an automated definition of GNSS-challenging volumes based on a georeferenced three-dimensional environment model, derivation of candidate obstacle-free paths between waypoints, waypoint assignment, and definition of time-tagged trajectories for all UAVs. Kim et al. [22] address the drone-aided delivery and pickup route planning problem under the background of healthcare services, a preprocessing algorithm, a Partition method, and a Lagrangian Relaxation (LR) method are developed as solution approaches to improve the computational performance. Lim et al. [23] presented a reliable parcel delivery schedule using drones, which considered the battery consumption rate as a function of payload in the operational planning optimization. Meanwhile, a variable preprocessing algorithm and primal and dual bound generation methods are proposed to improve the computational time.

Notably, most of the current research on multi-UAVs mission planning focuses on the integrated task assignment and path planning problem, which has led to a series of variants with additional attributes, such as multiple ground control stations, heterogeneous UAVs team, time window, task priority, heterogeneous target, etc. The sensors are assumed to be fixed on the UAV and cannot be replaced; thus, the sensor allocation problem is ignored. However, interchangeable sensor configuration and multiple sensors coordination are the essential features of the modern UAV platform. In general, little research considers sensor allocation, task assignment, and collision-free trajectory generation problems, simultaneously. In order to meet the application requirements of the real world, sensor allocation, task assignment, and collision-free path generation problems are integrated into this paper.

From the optimization algorithm’s perspective, to improve the quality of the solution and the optimized efficiency, many heuristic algorithms are presented to solve the multi-UAVs mission planning problems. Ramirez-Atencia et al. [24] presented a multi-objective genetic algorithm for solving the complex mission planning problem involving a team of UAVs and a group of ground control stations. The constraint satisfaction problem is merged into the mission planning framework to improve search efficiency. Then, Ramirez-Atencia et al. [25] proposed a weighted random generator incorporated into the mission planning framework to further improve the convergence rate. Wu et al. [26] presented a coupled and distributed mission planning framework for solving the integrated task assignment and path planning problem. The distributed genetic algorithm implements the mission planning framework to obtain a fast and feasible solution. Luo et al. [27] presented the multi-functions heterogeneous UAVs cooperative mission planning model, which restricts the heterogeneous UAV performed the task from the function level and function type.

Most of the mission planning framework is implemented by population-based optimization algorithms, such as genetic algorithm, particle swarm optimization, fruit fly optimization algorithm. These population-based optimization methods must repair many infeasible solutions in the iterative process [28], and the parameters are usually difficult to set. The variable neighborhood search (VNS) is an effective metaheuristic—several applications [29,30,31,32,33] in operations research have shown very competitive results. The heterogeneous multi-UAVs mission planning is an NP-hard combinatorial optimization problem with a large search space, challenging to be solved effectively by conventional methods. The variable neighborhood search algorithm optimizes the solution through neighborhood structure, which has high flexibility and is suitable for solving complex problems.

Our aim through this research is to explore an efficient mission planning framework for the heterogeneous multi-UAV systems to effectively deal with the integrated sensor allocation and path planning problem. Furthermore, we expect to develop an effective heuristic algorithm to improve the performance of the mission planning framework for large-sized problems. The main contributions and innovations of this paper are as follows:

(1) We build an integrated sensor allocation and path planning model, which is associated with three subproblems: Sensor allocation, task assignment, and collision-free path generation, simultaneously. Meanwhile, the model has a certain practical significance for cooperative reconnaissance, sensor information collection, and continuous monitoring missions in the real world, etc.

(2) Depending on the natural complexity of the problem, an integrated mission planning framework based on a novel two-level adaptive variable neighborhood search algorithm is proposed. The first-level is devoted to planning a reasonable airborne sensor allocation plan, and the second-level aims to optimize the collision-free path of the heterogeneous multi-UAVs system.

(3) The size of the integrated sensor allocation and path planning problem is determined by many factors: The number of heterogeneous UAVs, tasks, and sensors, etc. Due to there are many factors affecting the problem’s size, the size of the problem is usually large. In order to improve the optimization efficiency of the mission planning framework for large-sized problems. An adaptive mechanism is proposed to guide the search direction intelligently. Detailed simulations are given to demonstrate the effectiveness of the proposed framework.

The remainder of this paper is organized as follows. Problem formulation is given in Section 2. A brief introduction of the conventional Variable Neighborhood Search Algorithm is presented in Section 3. The integrated mission planning framework adopted in this paper is proposed in Section 4. Our method is comparatively simulated in Section 5. The Limitation analysis is shown in Section 6. In the end, conclusions are given in Section 7.

## 2. Problem Formulation

In this section, the integrated sensor allocation and path planning problem for a heterogeneous multi-UAVs system is presented. The problem is considered for mission scenarios involving a group of tasks that need to be performed by a heterogeneous multi-UAVs system in an environment with obstacles. Meanwhile, a group of interchangeable sensors is available at the base. Due to the task’s profit is related to the sensors loaded by the UAV performing the task. Therefore, the optimal objective of the problem is to maximize the total profit of the tasks and minimize the total path length of the heterogeneous multi-UAVs system. The integrated airborne sensor allocation and path planning problem is formulated as a mixed integer linear programming model, which is based on the outstanding work of Warrier [34] and Mufalli et al. [18].

### 2.1. Parameter Definitions

Let T={0,…,N} be the set of task locations required to be visited by a heterogeneous multi-UAVs system and 0 represents the base location, and each task i∈T must be performed only once. Let U={1,…,M} be the M-sized set of heterogeneous UAVs involving in the mission scenario, the heterogeneity of the UAVs is reflected in the upper load limit and kinematics characteristics. Let $T_{h}$ be the maximum travel distance of UAV h∈U. Note that the sensor loaded by the UAV h will reduce the maximum travel distance. The reduced travel distance is proportional to the weight of the sensor. Let $\varsigma_{h}$ represents the maximum loading weight of UAV h. Meanwhile, each UAV may be loaded with multiple sensors. Let $\tau_{h}$ be the maximum number of sensors that can be loaded by the UAV h. Let S={1,…,Z} be the Z-sized set of available sensors at the base and $Q_{s}$ be the quantity of sensor s∈S available at base. As mentioned above, the weight of the sensor will reduce the maximum travel distance of the UAVs. Let $w_{s}$ be the weight of sensor s and $C_{s}$ denotes the reduced travel distance when sensor s is loaded. Note that the task profit is no-static, which is associated with the sensors loaded by the UAV performing the task. Let $R_{js}$ be the task profit when the UAV is loaded with sensor s to perform the task j.

In this problem, the UAVs are required to fly over the tasks’ locations to complete them. For the sake of simplicity, we assume that (1) the UAVs flight can be maintained at a given altitude; (2) The interference of wind is ignored; (3) The flying altitude of UAVs is different, so the problem of collision between UAVs is ignored; (4) The airspeed of each UAV is constant.

The kinematics model of UAVs is expressed by the following formula:

(1) $$\dot{x}=v_{u}\cos\psi$$

(2) $$\dot{y}=v_{u}\sin\psi$$

(3) $$\dot{\psi }=c/\rho_{min}$$

where x and y are the Cartesian coordinates of UAVs. $v_{u}$ is the cruise velocity. ψ is the heading angle. c is the steering command, –1≤c≤1. $\rho_{min}$ is the minimum turning radius. Let $X_{obs}$ represents the obstacle region in the mission scenario. Suppose that $\Sigma_{h}=\left\{\sigma_{1},\ldots ,\sigma_{k}\right\}$ represents the permutation of the locations labels, that need to be visited by the UAV h, and $\pi_{\Sigma }^{h}$ be the collision-free path to visit the tasks’ locations associated with $\Sigma_{h}$. Let L(.) is the collision-free path length calculation function, the collision-free path length between i∈T and j∈T can be represented by L(i,j).

### 2.2. Combinatorial Optimization Problem

The integrated sensor allocation and path planning model is expressed by the following optimization problem:

(4) $$Maximize\;\overset{M}{\underset{h=1}{\sum }}\overset{N}{\underset{j=0}{\sum }}\overset{Z}{\underset{s=1}{\sum }}R_{js}z_{jsh}-\lambda \overset{M}{\underset{h=1}{\sum }}\overset{N}{\underset{j:j\neq i}{\sum }}\overset{N}{\underset{i=0}{\sum }}L\left(i,j\right)y_{ijh}$$

(5) $$Subject\;to:\;\begin{array}{cc} \overset{Z}{\underset{s=1}{\sum }}f_{hs}\leq \tau_{h} & \forall h\in U \end{array}$$

(6) $$\begin{array}{cc} \overset{M}{\underset{h=1}{\sum }}f_{hs}\leq Q_{s} & \forall s \end{array}\in S$$

(7) $$\begin{array}{cc} \overset{N}{\underset{j=0,i\neq j}{\sum }}y_{ijh}\leq 1 & \forall i\in T,h\in U \end{array}$$

(8) $$\overset{N}{\underset{j=0;j\neq i}{\sum }}y_{ijh}-\overset{N}{\underset{j=0;j\neq i}{\sum }}y_{jih}=0\;\;\forall h\in U$$

(9) $$\begin{array}{cc} \overset{S}{\underset{s=1}{\sum }}f_{hs}w_{s}\leq \varsigma_{h} & \forall h\in U \end{array}$$

(10) $$\overset{N}{\underset{j:j\neq i}{\sum }}\overset{N}{\underset{i=0}{\sum }}L\left(i,j\right)y_{ijg}\leq T_{h}-\overset{S}{\underset{s=1}{\sum }}C_{s}f_{hs}\;\;\forall h\in U$$

(11) $$\begin{array}{cc} \overset{N}{\underset{i=0}{\sum }}y_{i0h}=1 & \forall h\in U \end{array}$$

(12) $$\begin{array}{cc} \overset{N}{\underset{i=0}{\sum }}y_{0ih}=1 & \forall h\in U \end{array}$$

(13) $$\pi_{\Sigma }^{h}\cap X_{obs}=\emptyset \;\;\forall h\in U$$

(14) $$\rho_{h}\geq \rho_{min}^{h}\;\forall h\in U$$

(15) $$f_{hs},y_{ijh},z_{jsh}\in \left\{0,1\right\}$$

where $z_{jsh}$, $y_{ijh}$, and $f_{hs}$ are binary decision variables. $z_{jsh}$ equals one if UAV h is equipped with sensor s to perform task j, and $y_{ijh}$ equals one if UAV h travels from the task location of task i to the task location of task j. $f_{hs}$ equals one if UAV h is equipped with sensor s. Note that λ is a weight factor, which is used to balance the optimization objective. The first term of the objective function (1) aims to maximize the total task profit, while the second term attempts to minimize travel costs for the heterogeneous multi-UAVs system.

Constraint (5) ensures that the number of sensors equipped with UAV does not exceed the maximum number of sensors that UAV can be equipped with. Constraint (6) guarantees that the total number of any sensors equipped by the UAV does not exceed the number of sensors available at the base. Constraint (7) and (8) ensure that each task is only performed once. Constraint (9) makes sure that the weight of the sensor loaded on the UAV does not exceed the maximum load of the UAV.

Constraint (10) guarantees that the travel distance of the UAV does not exceed its maximum travel distance. Constraint (11) and (12) ensure that all UAVs begin and end their path at the base. Constraint (13) makes sure that the path of the UAVs will not collide with obstacles. Constraint (14) is the kinematic constraint of UAV, which ensures that the turning radius of UAV is not less than its minimum turning radius. Constraint (15) is the binary constraint.

## 3. Brief Introduction of the Conventional Variable Neighborhood Search Algorithm

**Figure 1:**
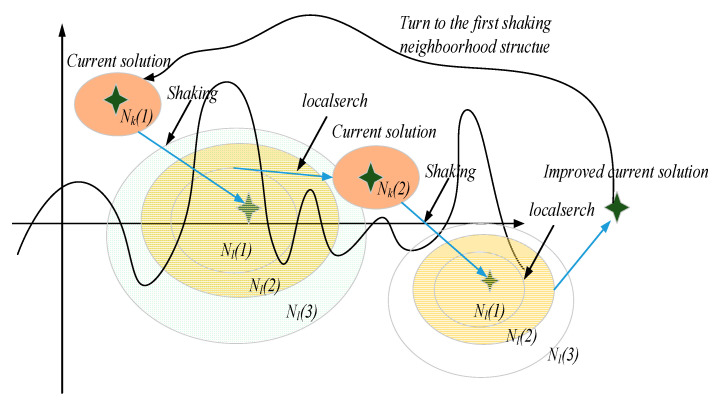
Conventional VNS concept diagram.

As an efficient meta-heuristic algorithm, VNS has been successfully applied in many fields. Unlike the most meta-heuristic algorithm, the VNS uses the neighborhood structure to search the solution space alternately. The concept diagram of the conventional VNS is shown in Figure 1. The first shaking neighborhood structure will first change the current solution, and then the newly generated solution will be systematically searched by the local search heuristic. If the current solution is not improved, the next shaking neighborhood structure will change it. If the current solution is improved, it will turn to the first shaking neighborhood structure.

The VNS is mainly composed of two essential procedures: (1) Shaking procedure, (2) local search. The shaking procedure is used to change the current optimal solution by shaking neighborhood structures to prevent the solution from falling into the local optimum. The local search algorithm will change the solution systematically by several local search neighborhood structures. The procedure of conventional VNS is shown in Algorithm 1.
**Algorithm 1.** The Procedure of the Conventional VNS1:Let s$\;\mathrm{be}\;\mathrm{the}\;\mathrm{initial}\;\mathrm{solution},\;N_{k}$ be the shaking neighborhood structures, $k=1,\ldots ,k_{max}$ and $N_{l}$ be the local search $\mathrm{neighborhood}\;\mathrm{structures},\;l=1,\ldots ,l_{max}$. Set k=1.2:**Repeat**3: *{Shaking procedure}*4: $\;s^{\prime }=Shaking\left(s,N_{k}\right)$ //*Shaking procedure*5: *{Local Search}*6: **for**l=1**to**$l_{max}$**do** //*Local search*7: $\;\;\;s^{\prime \prime }=LocalSearch\left(s^{\prime },N_{l}\right)$*//perform local search on*$s^{\prime }$*with neighborhood structure* $N_{l}$8:   **if** $f\left(s^{\prime \prime }\right)>f\left(s^{\prime }\right)$*//checking if*$s^{\prime }$*is improved*9:$\;\;s^{\prime }=s^{\prime \prime }$10:  l=1*//turn to the first neighborhood structure for local search*11:   **else**12:  l=l+1*//turn to the next neighborhood structure for local search*13: **end for**14: *{Accept decision}*15: **if** $f\left(s^{\prime }\right)>f\left(s\right)$*//checking if*s is improved16:$\;\;\;s=s^{\prime }$17:   k=1*//turn to the first neighborhood structure for shaking*18: **else**19:   k=k+1*//turn to the next neighborhood structure for shaking*20: **end if**21:**Until** the upper limit T of time or the maximum number of iterations $Z_{max}$ is reached

## 4. Integrated Mission Planning Framework Base on the Two-Level Adaptive Variable Neighborhood Search Algorithm

### 4.1. Overview of the Integrated Mission Planning Framework

Depending on the problem’s complexity, we propose an integrated mission planning framework base on the two-level adaptive variable neighborhood search algorithm. The first-level focuses on allocating sensors for the heterogeneous multi-UAVs system to maximize the total task profit. The second-level aims to generate the sequence of tasks assigned to each vehicle within the heterogeneous multi-UAV system to minimize travel costs. Collision-free path definition between a couple of consecutive tasks as detailed in Section 4.5. The structure of the mission planning framework based on the adaptive two-level variable neighborhood search algorithm is shown in Algorithm 2 in pseudo-code.
**Algorithm 2.** The Structure of the Mission Planning Framework Based on the Adaptive Two-Level Variable Neighborhood Search Algorithm1:Set the shaking neighborhoods structures of the first-level $N_{k}^{1}$ with $k=1,\ldots ,k_{max}$ and the shaking neighborhoods structures of the second-level $N_{d}^{2}$ with $d=1,\ldots ,d_{max}$. Set the local search neighborhood structures of the first-level $N_{l}^{1}$ with $l=1,\ldots ,l_{max}$, the local search neighborhood structures of the second-level $N_{w}^{2}$ with $w=1,\ldots ,w_{max}$. Let i=0, l=1 and d=1.2:*{Initialization phase}*3:s=GenInitialSolution() //generate an initial solution4:**Repeat**5: *{First-Level Adaptive Shaking: Sensor allocation} //first-level*6:$\;k=AdaptiveShaking\left(N_{k}^{1}\right)$*//Adaptive select the shaking neighborhood structure, the UAVs, and sensors for shaking*7$\;s_{1}=FirstLevelShaking\left(s,N_{k}^{1}\right)$*//generate the shaking solution according to shaking neighborhoods structure*$N_{k}^{1}$.8: *{First-Level Local Search Phase: Sensor allocation}*9: ***while*** $l<l_{max}$10:$\;\;\;s_{2}=FistLevelLocalSearch\left(s_{1},N_{l}^{1}\right)$*//perform local search on*$s_{1}$*according to local search neighborhoods structure*$N_{l}^{1}$.11:    ***while***$d<d_{max}$*//second-level*12:    *{Second-Level Shaking Phase: Route Planning}*13:$\;\;\;\;s_{3}=SecondLevelShaking\left(s_{2},N_{d}^{2}\right)$*//generate the shaking solution of the second-level base on the shaking neighborhoods structure*$N_{d}^{2}$.14:    *{Second-Level Local Search Phase: Route Planning}*15:    ***while***$w<w_{max}$16:$\;\;\;s_{4}=SecondLevelLocalSearch\left(s_{3},N_{w}^{2}\right)$*//perform local search on*$s_{3}$*according to local search neighborhoods structure*$N_{w}^{2}$.17:   ***if***$\;f\left(s_{4}\right)>f\left(s_{2}\right)$18:$\;\;\;\;s_{2}=s_{4}$19:    d=120:   ***else***21:    d=d+122:   ***end if***23:    ***end while***24:    ***end while***25: *{Acceptance decision}*26:    *if*$f\left(s_{2}\right)>f\left(s\right)$*or*$s_{2}$*is accepted*27:$\;\;\;s=s_{2}$28:   l=129:    *else*30:   l=l+131:    ***end if***32: ***end while***33:**Until** the upper limit T of time or a given number Iter of iterations without improvement is reached

The adaptive two-level variable neighborhood search algorithm starts with an initial solution s. The initial solution is generated by the GenInitialSolution() function, which is detailed in Section 4.2. Subsequently, the main procedure is repeated until a given stopping criterion is met. In the main procedure, the shaking neighborhood structure, the UAVs, and the sensors involving in the shaking procedure of the first-level is adaptively selected based on the $AdaptiveShaking\left(N_{k}^{1}\right)$ procedure to guide the search direction Then, given the current solution s, the FirstLevelShaking procedure is applied, which generates a new solution $s_{1}$ in the k-th shaking neighborhood structure for the first-level $N_{k}^{1}$. The $AdaptiveShaking\left(N_{k}^{1}\right)$ and FirstLevelShaking procedures are detailed in Section 4.3.1 and Section 4.3.2. In the next step, the $FistLevelLocalSearch\left(s_{1},N_{l}^{1}\right)$ procedure is performed and the solution $s_{2}$ is generated, which is detailed in Section 4.3.3. The second-level variable neighborhood search procedure is embedded in the local search procedure of the first-level. The main components of the second-level variable neighborhood search also consist of a shaking procedure and a local search procedure, which are detailed in Section 4.4. Notably, the second-level attempts to optimize the target location visited sequence of each UAV and redistributed the target location responsible for the routes. In the process of adjusting the visit order of target locations, it is a critical procedure to calculate the collision-free trajectory length of UAVs, which is detailed in Section 4.5. The local search step of the first-level and the second-level variable neighborhood search algorithm jointly determine the local optimum solution $s_{2}$. If the solution $s_{2}$ improves the solution s or the solution $s_{2}$ is accepted by a specify acceptance mechanism, then $s_{2}$ replaces s, the acceptance mechanism is detailed in Section 4.6.

### 4.2. Initial Solution

The initial solution for the adaptive two-level variable neighborhood search algorithm is conducted by a two-phase greedy heuristic. The first phase aims to generate the path for each UAV by the savings algorithm. The saving algorithm was proposed by Clarke and Wright, which is a classical algorithm in the field of logistics and transportation. The saving algorithm is mainly used to optimize the team’s driving distance in multivehicle transportation. Its principle is to continuously merge paths to shorten the total driving distance. For more details about the saving algorithm, please refer to the literature [35]. The second phase randomly assign sensors to the heterogeneous multi-UAV system.

### 4.3. Adaptive Shaking and Local Search of the First-Level

In the shaking procedure of the first-level, the heterogeneous UAVs and the sensors involving in the shaking procedure are determined by several adaptive rules. Then, the shaking solution of the first-level is generated based on the predefined shaking neighborhood structures. The adaptive mechanism adopted in the first-level shaking procedure will guide the following search direction to improve the algorithm’s convergence efficiency.

#### 4.3.1. Shaking Neighborhood Structures of the First-Level

**Figure 2:**
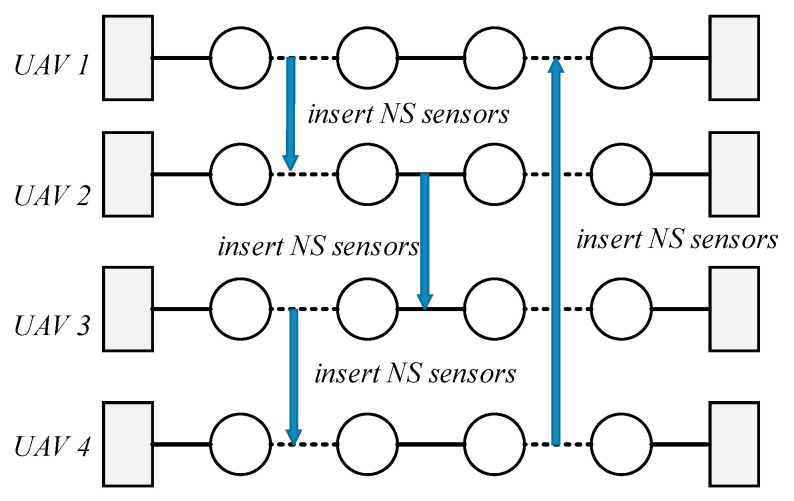
An example of a sensor cyclic exchange operator.

The neighborhood structures are directly related to the search range of the algorithm, which will greatly affect the algorithm’s convergence and the optimization quality of the solution. In the first-level shaking procedure, the sensors loaded by the heterogeneous multi-UAVs system will be optimized to maximize the total task profit. Let $S_{K}=\left\{s_{1},\ldots ,s_{M}\right\}$ be the sensors set loaded by the heterogeneous multi-UAVs system in the current solution. Let $s_{k}$ represents the sensors set loaded by UAV k∈K, $s_{k}\in S_{K}$. The total number of sensors loaded by the UAV k is $\left|s_{k}\right|$ and the i-th sensor loaded by the UAV k is denoted by $e_{i}^{k}$. Thus, the sensors set loaded by the UAV k can be expressed by $s_{k}=\left\{e_{1}^{k},e_{2}^{k},\ldots ,e_{\left|s_{k}\right|}^{k}\right\}$. The remaining sensors in the base are uniformly denoted as a set $Z=\left\{z_{1},\ldots ,z_{n}\right\}$. The shaking neighborhood structures are shown in Table 1. The shaking neighborhood operators adopted in the first-level is as follows:

1. Sensor cyclic exchange operator: The sensor cyclic exchange operator will exchange the sensors currently loaded by the heterogeneous multi-UAVs system cyclically. An example of a sensor cyclic exchange operator is shown in Figure 2. Two critical parameters are involved in the sensor cyclic exchange operator: The number of heterogeneous UAVs involved in the shaking procedure NU and the maximum number of sensors to be exchanged NS.

For each heterogeneous UAV k involved in the shaking procedure, a subset of sensors is selected from $s_{k}\in S_{K}$. Then, the sensors will be inserted to another heterogeneous UAV k+1 involving in the shaking procedure. Note that, the constraints (5), (6), (9), and (10) cannot be violated in this neighborhood structure.

**Figure 3:**
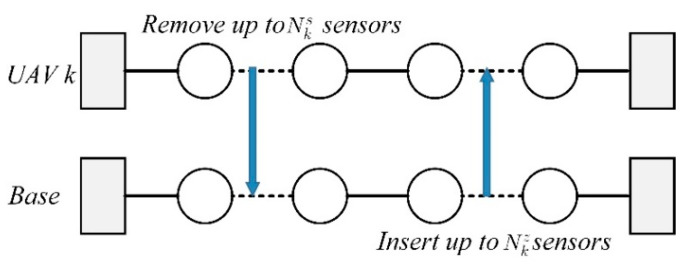
An example of an unbalanced exchange operator.

2. Unbalanced exchange operator: For the heterogeneous UAV k∈K, the unbalanced delete & insert operator will delete up to $N_{k}^{s}$ sensors from the sensor set $s_{k}\in S_{K}$. Then, up to $N_{k}^{z}$ sensors will be selected from the sensor set Z and inserted in the sensor set $s_{k}$. Notice that the number of sensors deleted from the sensor set $s_{k}$ maybe not equal to the number of sensors inserted. Similarly, the constraints (5), (6), (9) and (10) cannot be violated in this neighborhood structure. An example of an unbalanced exchange operator is shown in Figure 3.

#### 4.3.2. Adaptive Mechanism

Selecting suitable heterogeneous UAVs and sensors to participate in the shaking procedure can improve the algorithm’s efficiency in searching for high-quality solutions. Thus, we develop several adaptive selection methods to determine the heterogeneous UAVs and sensors involving the first-level shaking procedure. The selection method includes the selection method of heterogeneous UAV and the selection method of sensors.

Heterogeneous UAV selection method. The heterogeneous UAV selection method selects NU heterogeneous UAVs that be involved in the sensor cyclic exchange operator. The selection method is performed in two steps: (1) Select the first heterogeneous UAV to participate in the shaking procedure through the following three selection mechanisms. (2) Randomly select the remaining NU-1 heterogeneous UAVs. The three methods are used to select the first heterogeneous UAV is expressed as follow:

1. Random: The heterogeneous UAVs in the current solution have the same probability to be selected.
2. Largest remaining load: The selection probability of a heterogeneous UAV is proportional to its remaining load. The goal is to optimize the sensor configuration for the UAV with the largest remaining load.
3. Maximum number of visit targets: The probability of the heterogeneous UAV being selected is proportional to the number of visit targets. The goal is to optimize the sensor configuration of the heterogeneous UAV with the maximum number of visit targets.

Sensor selection method. After the first heterogeneous UAV to be involved in the sensor cyclic exchange operator is selected. The sensors of the heterogeneous UAV to be involved in the sensor cyclic exchange operator will be determined. The three methods are used to select the sensors loaded by the selected heterogeneous UAV is expressed as follow:

1. Random: All the sensors involved loaded by the selected heterogeneous UAV have the same probability of being selected.
2. Total task profit: For the selected UAV, the selection probability of the sensor is inversely proportional to the total task profit. The goal is to assign sensors with higher total task profit to the selected UAV.
3. Travel distance reduction: For the selected UAV, the selection probability of the sensor is proportional to the travel distance reduction. The goal is to remove the sensor with higher travel distance reduction to increase the maximum travel distance of the selected UAV.

The selection method will significantly affect the efficiency of the search process. In the first-level shaking procedure, we design an adaptive mechanism to realize the selection method’s intelligent decision. The roulette wheel selection procedure is adopted to determine the heterogeneous UAV and sensor selection methods. In the algorithm’s initial stage, each selection method’s selected probability is set to the same value. With the progress of the search process, the selected probability of each selection method is gradually updated. Specifically, each method’s selected probability is uniformly updated after γ iterations of the two-level mission planning framework based on a scoring system. The scoring system has the following statistical rules: (1) A score of nine will be added to a selection method if the overall optimal solution is obtained after applying the method. (2) A score of three will be added to a selection method if the current solution is improved. (3) A score of one will be added to a selection method if the new solution is worse than the current solution, but the solution is accepted base on the acceptance criterion. Let η be the total number of the selection method and $\delta_{i}$ be the current score of the method i, 1≤i≤η. Notice that each selection methods i=1,…,η is associated with a weight coefficient $\omega_{i}$ and the probability of the method i being selected is $\omega_{i}/\overset{\eta }{\underset{j=1}{\sum }}\omega_{j}$. Suppose that as of the last score update, the method i has been selected $\alpha_{i}$ times, then the new score of the method i is updated by $\omega_{i}\left(1-\rho \right)+\rho \left(\delta_{i}/\alpha_{i}\right)$, where ρ∈[0,1] is a system parameter for controlling the trend of score update.

#### 4.3.3. Local Search Neighborhood Structures of the First-Level

The solution generated by the shaking procedure is then improved by the local search step. The local search is implemented by a set of neighborhood structures. Five neighborhood structures involving in the local search step is defined as follow:

1. Insertion operator: The operator will randomly select a sensor from the sensor set Z and insert it into the sensor set $s_{k}$ loaded on a UAV k∈K.
2. Exchange operator: The operator will randomly exchange a sensor $e_{i}^{k}\in s_{k}$ with a sensor $e_{j}^{l}\in s_{l}$, where $1\leq i\leq \left|s_{k}\right|$, $1\leq j\leq \left|s_{l}\right|$, $s_{k},s_{l}\in S$ and k≠l.
3. 2-exchange operator: Similar to the exchange operator, the 2-exchange will randomly exchange two sensors in $s_{k}$ with two sensors in $s_{l}$, where $s_{k},s_{l}\in S$ and k≠l.
4. 1-deletion-1-insertion operator: The operator will delete a sensor from $s_{k}$ and insert another sensor from Z to $s_{k}$.
5. 2-deletion-2-insertion operator: The operator will delete two sensors from $s_{k}$ and add them to Z. Then, two sensors from Z is inserted to $s_{k}$.

### 4.4. Shaking and Local Search of the Second-Level

The second-level of the adaptive variable neighborhood search aims to minimize travel costs for the heterogeneous multi-UAVs system. As shown in Algorithm 2, the second-level variable neighborhood search is embedded in the first-level’s local search step to improve the solution generated by the first-level. The shaking and local search neighborhood structures are given in this section.

**Figure 4:**
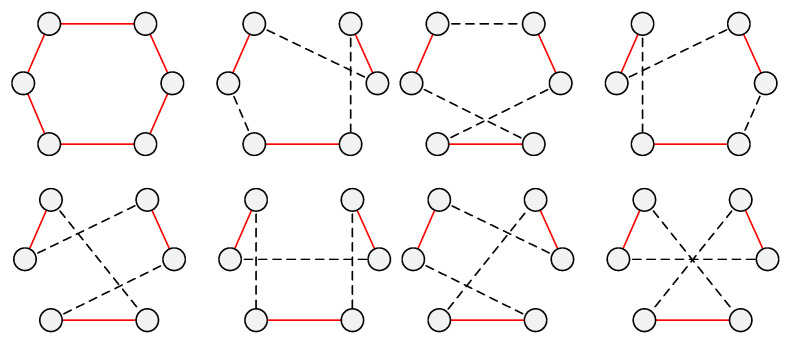
Eight forms of the intraroute 3-opt operator.

Shaking procedure. Similar to the first-level perturbation procedure, the second-level perturbation procedure is to prevent the solution from falling into the local optimum. The shaking neighborhood structures are designed as follow:

1. Intraroute 3-opt: The interroute 3-opt operator is an extension of the 2-opt operator, which is used to change the visit sequence of the nodes in a route. As shown in Figure 4, eight forms of the intraroute 3-opt operator are given, and one of them will be randomly selected.
2. Interroute 2-exchange: The interroute 2-exchange operator randomly exchanges two pairs of nodes in two different routes.

**Figure 5:**
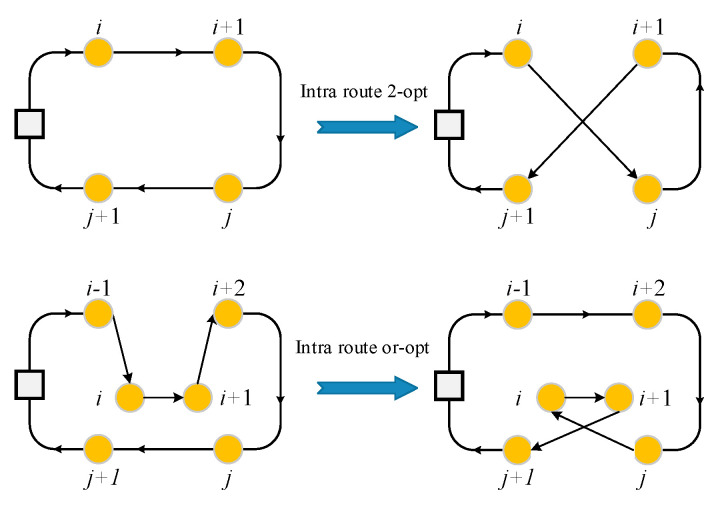
An example of intraroute 2-opt and intraroute or-opt.

**Figure 6:**
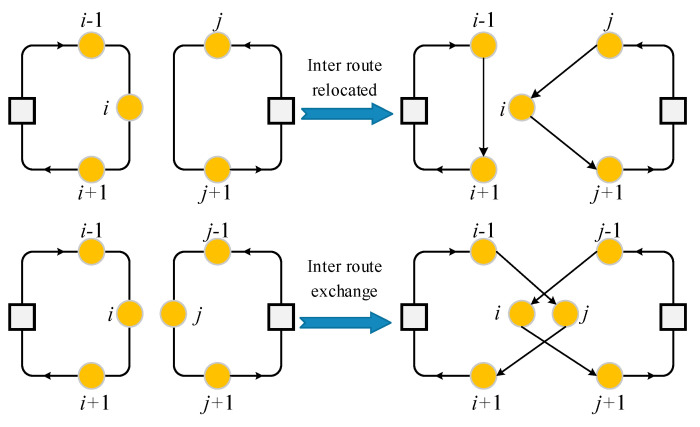
An example of interroute exchange and interroute relocated.

The solution generated by the second-level’s shaking produce is then input to the second-level’s local search. The local search aims to find an improved solution by changing the visit sequence of nodes in the same route and exchanging the visit nodes between different routes. Four local search neighborhoods structures are given as follow:

1. Intraroute 2-opt: The intraroute 2-opt operator will reverse the nodes visit sequence of a sub-route.
2. Intraroute or-opt: The intraroute or-opt operator relocates several consecutive nodes in a route. An example of intraroute 2-opt and intraroute or-opt are shown in Figure 5.

3. Interroute exchange: The interroute exchange operator randomly exchanges two nodes in the same route.
4. Interroute relocated: The interroute relocated randomly delete a node from a route and insert it to another route. An example of interroute exchange and interroute relocated is shown in Figure 6.

### 4.5. Collision-Free Path Planning

The optimization objective is closely related to the flight path length of the heterogeneous UAV system. To calculate the path length of a heterogeneous multi-UAV system, we must first obtain the collision-free path of each UAV in space. Note that the collision-free path needs to satisfy both kinematic and collision-free constraints of heterogeneous UAVs. However, a large number of solutions are generated in the two-level mission planning framework. In order to improve the optimization efficiency, the constraints that the UAV needs to satisfy are relaxed during the iteration process. That is, in the iterative process, the collision-free route length of the UAV to visit each node is evaluated, while the kinematic constraints of the UAV are ignored. Specifically, the collision-free path planning process is separated into multiple phases, which are presented as follows:

1. Map preprocessing. In this stage, the continuous space is discretized based on the target location and the fixed point of the obstacle. Note that this procedure is only evaluated once when the objective function value is calculated for the first time. The visibility map is adopted in this stage to discretize the space efficiently.
2. Route assessment. The route assessment stage is used to calculate the objective value of the solutions in the iterative process. In this stage, the A * algorithm is adopted to generate the shortest polyline path connecting the UAV’s initial location and target location sequence.
3. Path smoothing. As a postprocessing stage, this stage is used to optimize the path of the final solution. The polyline path of the UAV mentioned above is smoothed to generate a collision-free trajectory satisfying the kinematic constraints of the UAV.

In order to reduce the calculation cost, the collision-free path between any two locations is precalculated and stored in the form of distance matrix $D_{M}={\left[d_{0}^{0},\ldots ,d_{0}^{N};d_{1}^{0},\ldots ,d_{1}^{N};\ldots d_{N}^{0},\ldots ,d_{N}^{N}\right]}_{\left(N+1\right)\times \left(N+1\right)}$. The second-level adaptive variable neighborhood search algorithm obtains the collision-free path length between any two task locations by querying the distance matrix.

### 4.6. Acceptance Decision

Acceptance Decision. The acceptance decision process is executed after each round of the second-level local search procedure. The new solution generated by the second-level local search procedure $s_{2}$ will be compared with the current solution s. Once the new solution $s_{2}$ is accepted, the solution $s_{2}$ will replace the solution s become the current solution. In order to improve the diversity of solutions, the acceptance decision process incorporates the solution acceptance mechanism of the simulated annealing algorithm. Specifically, the improved solution will always be accepted by the algorithm, that is $f\left(s_{2}\right)>f\left(s\right)$. Notice that non-improving solution will also be accepted with a certain probability $e^{-\left(f\left(s\right)-f\left(s_{2}\right)\right)/\theta }$, where θ is the temperature parameter and f(.) is the objective value of the objective function. The initial value of θ is set to $\theta_{0}$, which decreases with each iteration of the algorithm with a coefficient $\theta^{-}$. If the solution cannot be improved after v iterations, then θ will be reset to zero.

## 5. Simulation and Discussion

In this section, the proposed integrated mission planning framework for heterogeneous multi-UAV systems was implemented. To verify the feasibility of the mission planning framework, we designed a simple mission scenario and optimized it through the mission planning framework. Meanwhile, we analyzed the solution generated by the mission planning framework to ensure that the constraints were not violated. To test the performance of the mission planning framework, the algorithm is compared with other algorithms. The simulation hardware environment is Intel Core (TM) i7-6500u CPU @ 2.50 GHz notebook computer, and the programming environment is visual studio 2015.

### 5.1. *Feasibility Verification*

#### 5.1.1. Test Scenario and Parameter Settings

This section gives a specific test scenario to verify the feasibility of the mission planning framework’s solution. The scenario involves four heterogeneous UAVs and three types of sensors. The attributes of the sensors and UAVs are shown in Table 2 and Table 3, respectively. In this scenario, four obstacles are set up to test whether the heterogeneous Multi-UAVs system violates the collision-free and kinematic constraints. In this scenario, 31 tasks are given, and the sensors—task benefit matrix is shown in Table 4. The maximum number of iterations for the two-level adaptive variable neighborhood search algorithm was set to 30,000 and terminated when no improvement was found in 1000 iterations. The parameter $\theta_{0}$ is related to the convergence effect of the algorithm. A larger value can expand the search space of the solution, but cause a slower convergence rate. Further, the coefficient $\theta^{-}$ and the number of no improving iterations v also directly impact the convergence effect. Through numerical experiments, we set $\theta_{0}=18.7$, $\theta^{-}=0.05\%$, and v=180. Further, the weight factor λ is set to 0.1. The mission area is set to a square area of 2000 × 2000 $m^{2}$.

#### 5.1.2. Feasibility Analysis

**Figure 7:**
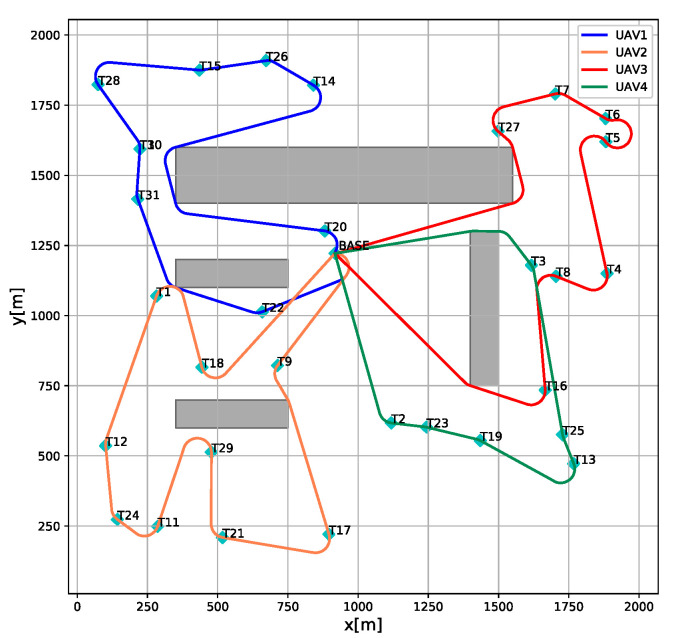
The task execution paths of the heterogeneous unmanned system.

The result of sensor allocation is shown in Table 5. From the calculation results, it can be found that all constraints have not been violated. The task execution paths of the heterogeneous multi-UAVs system are shown in Figure 7. Notice that the collision-free constraints of the UAVs are respected. Meanwhile, the paths of the heterogeneous multi-UAVs system satisfy the constraints of kinematics. Therefore, the feasibility of the solution generated by the mission planning framework is verified.

### 5.2. Numerical Comparisons

#### 5.2.1. Experimental Conditions

To verify the integrated mission planning framework’s performance based on the two-level adaptive variable neighborhood search algorithm. We developed nine mission scenarios as evaluation benchmarks. The summary of the test case is given in Table 6. The locations of the tasks were randomly distributed in the mission area. The cruise speed of heterogeneous UAVs is uniformly set to 20 m/s, and the minimum turning radius is randomly selected from 30 m to 45 m. Meanwhile, the probability that a task has benefited from each sensor is set evenly. The task benefit value is set based on the distribution, benefit range 0~50 with the probability 0.1, 50~100 with the probability 0.2, 100~150 with the probability 0.3, and 150~200 with the probability 0.4. The upper load limit of the first UAV in each test case is set to 150, and the subsequent UAVs are increased by 30. The first type of sensor in each test case is set to 50, and the subsequent type of UAVs are increased by 25.

The case ID from 1~3 is considered the benchmark of small-sized problems, 4~6 is considered the benchmark of medium-sized problems, and 7~9 is considered the benchmark of large-sized problems. The value of maximum unloads travel distance for each UAV is set based on the number of UAVs in the mission scenario. For the small-sized problems, the maximum unload travel distance for each UAV is randomly selected from 10,000~15,000 m, 15,000~25,000 m for the medium-sized problems, and 25,000~35,000 m for the large-sized problems. The weight factor is set to 0.5 for the small-sized problems, 0.2 for the medium-sized problems, and 0.1 for the large-sized problems.

#### 5.2.2. Monte Carlo Study

To test the mission planning framework’s performance in the small-sized problems, medium-sized problems, and large-sized problems. We have developed another two variable neighborhood search algorithms for comparative analysis. They are the two-level variable neighborhood search algorithm without the adaptive mechanism (TLVNS) and the two-level variable neighborhood search algorithm with a random selection strategy (TLVNS_RSS).

For the TLVNS, compared to TLAVNS, its adaptive mechanism is removed, and the shaking neighborhood structures: ID 1, 5, 6, 9, 12 in Table 1 is performed sequentially, as did in conventional VNS. For the TLVNS_RSS, the shaking neighborhood structures are randomly selected from ID 1 ~ 13 in Table 1. The number of the neighborhood is set to 5. In order to compare the algorithms effectively, Monte Carlo simulation is introduced. The TLVNS, TLVNS_RSS, and TLAVNS algorithms are run 2000 times, respectively, for each test case in Table 6.

**Figure 8:**
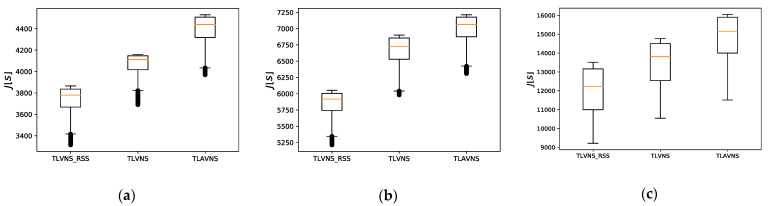

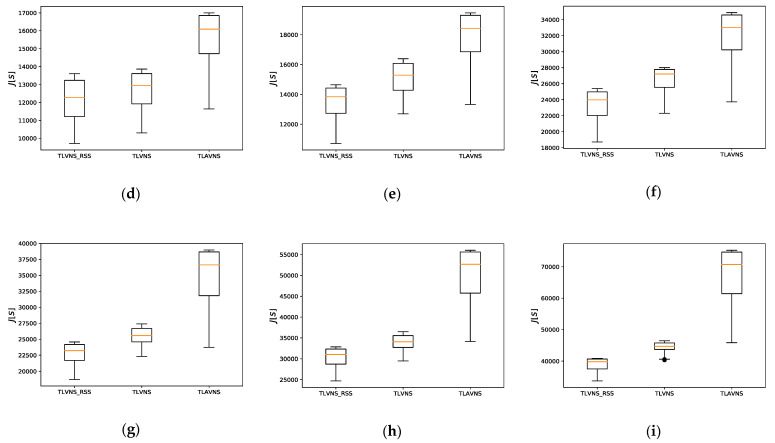
(**a**–**c**) The distribution of solutions for the small sized problem; (**d**–**f**) The distribution of solutions for the medium sized problem; (**g**–**i**) The distribution of solutions for the large sized problem.

The distribution of solutions for the small-sized problem is shown in Figure 8a–c, corresponding to the case-1, case-2, and case 3 in Table 6. In the Monte Carlo simulations for the small-sized problems, the proposed TLAVNS can obtain better results than the TLVNS_RSS and TLVNS. However, the distribution of the results is similar, especially between TLAVNS and TLVNS. The distribution of solutions for the medium-sized problem is shown in Figure 8d–f, corresponding to the case-4, case-5, and case 6 in Table 6. In the Monte Carlo simulations for the medium-sized problems, compared with small problems, the TLAVNS algorithm has better performance in medium problems than the other two algorithms. Meanwhile, the solution space of the TLVNS_RSS and TLVNS becomes smaller because they cannot find the optimization direction accurately. The distribution of solutions for the large-sized problem is shown in Figure 8g–i, corresponding to the case-7, case-8, and case 9 in Table 6. In the Monte Carlo simulations for the large-sized problems, the results show that our proposed TLAVNS can effectively deal with large-sized problems, and the adaptive mechanism can effectively guide the search direction. The average calculation time of the TLVNS_RSS algorithm in all test cases is about 132.2 s. The average calculation time of the TLVNS algorithm in all test cases is about 121.6 s. And the average calculation time of the TLAVNS algorithm in all test cases is about 169.3 s. From the perspective of calculation cost, the average calculation time of the TLAVNS algorithm is slightly higher than the other two algorithms. However, the increase in computation time is not obvious. Compared with the improvement of optimization performance, the increase in computation time can be tolerated. In general, our integrated mission planning framework based on the adaptive two-level variable neighborhood search algorithm can effectively deal with integrated sensor allocation and path planning problems. Furthermore, the adaptive mechanism can effectively guide the solution’s search direction, which makes the mission planning framework have good performance in dealing with medium and large-scale problems.

## 6. Limitation Analysis

The integrated sensor assignment and path planning model proposed in this paper may have some limitations in the real world applications. These limitations may lead to the failure of mission planning. The main application limitations are as follows:

(1) Uncertain factors in the process of task execution may lead to the failure of the plan, such as wind interference, weather impact on the performance of the sensor, etc.

(2) In order to simplify the complexity of the problem, we assume that UAVs fly at different altitudes to avoid collision between UAVs. However, this may not meet the requirements of some real-world tasks.

## 7. Conclusions

This paper focused on the integrated sensor allocation and path planning problem for a heterogeneous multi-UAVs system with interchangeable sensors and no-static mission profit. We presented an integrated mission planning framework based on a two-level adaptive variable neighborhood search algorithm to address the mission planning problem. The first-level is devoted to solving the sensor allocation problem. The second-level is embedded in the first-level framework, and the second-level is used to solve the path planning problem. The adaptive mechanism is introduced into the mission planning framework, which effectively improves the performance for large-sized problems. Simulation results showed our mission planning framework can generate good-quality solutions for large-sized mission planning problems. In order to deal with the above problems, we will focus on environmental uncertainty and collision avoidance in future research. Meanwhile, we will optimize the mission planning framework to improve its operational efficiency.

## Figures and Tables

**Table 1:** The shaking neighborhood structures.

ID	Operator	NU ($N_{k}^{s}$)	NS ($N_{k}^{z}$)	ID	Operator	NU ($N_{k}^{s}$)	NS ($N_{k}^{z}$)
1	Unbalanced exchange operator	1	1	8	Sensor cyclic exchange	3	1
2	Unbalanced exchange operator	1	2	9	Sensor cyclic exchange	3	2
3	Unbalanced exchange operator	2	1	10	Sensor cyclic exchange	3	3
5	Sensor cyclic exchange	2	1	11	Sensor cyclic exchange	4	1
6	Sensor cyclic exchange	2	2	12	Sensor cyclic exchange	4	2
7	Sensor cyclic exchange	2	3	13	Sensor cyclic exchange	4	3

**Table 2:** Sensors attributes.

Sensor	Quantity	Weight	Travel Distance Reduction (m)
S1	3	125	2500
S2	6	100	2000
S3	4	75	1500

**Table 3:** UAVs attributes.

UAV	Load Limit	Travel Distance (m)	Cruising Speed (m/s)	Minimum Turning Radius (m)	Sensor Capacity
U1	200	11,000	20	45	2
U2	240	12,000	20	45	2
U3	175	10,000	20	45	2
U4	220	13,000	20	45	2

**Table 4:** Sensor-Task Benefit Matrix.

	T1-T3	T4-T6	T7-T9	T10-T12	T13-T15	T16-T18	T19-T21	T22-T25	T26-T28	T29-T31
**S1**	72	24	46	35	12	83	42	52	23	42
**S2**	98	92	88	62	45	67	57	84	46	54
**S3**	44	75	37	27	29	23	44	45	57	36

**Table 5:** Result of sensor allocation.

	Sensors #1	Sensors #2	Load Limit	Sensors Weight	Maximum Travel Distance	Task Profit	Travel Distance
UAV1	S2	S3	200	175	7500	764	3920.98
UAV2	S1	S2	240	225	7500	1129	4425.97
UAV3	S2	S3	175	175	6500	944	3955.63
UAV4	S2	S2	220	200	9000	1054	2882.15

**Table 6:** Summary of the test case.

Case ID	Task Quantity	UAV Quantity	Sensor Type	Sensor Quantity	UAV Sensor Capacity	Mission Area m^2^
1	20	3	6	24	3	2000 × 2000
2	30	4	6	40	3	2000 × 2000
3	40	5	6	60	4	2000 × 2000
4	50	4	8	32	3	4000 × 4000
5	60	5	8	50	3	4000 × 4000
6	70	6	8	64	4	4000 × 4000
7	80	6	12	48	4	6000 × 6000
8	90	7	12	70	5	6000 × 6000
9	100	8	12	96	6	6000 × 6000

## Data Availability

Not applicable.
